# Artificial Small Molecules as Cofactors and Biomacromolecular Building Blocks in Synthetic Biology: Design, Synthesis, Applications, and Challenges

**DOI:** 10.3390/molecules28155850

**Published:** 2023-08-03

**Authors:** Fenghua Liu, Lingling He, Sheng Dong, Jinsong Xuan, Qiu Cui, Yingang Feng

**Affiliations:** 1 CAS Key Laboratory of Biofuels, Shandong Provincial Key Laboratory of Synthetic Biology, Qingdao Institute of Bioenergy and Bioprocess Technology, Chinese Academy of Sciences, 189 Songling Road, Qingdao 266101, China; 2 Shandong Energy Institute, 189 Songling Road, Qingdao 266101, China; 3 Qingdao New Energy Shandong Laboratory, 189 Songling Road, Qingdao 266101, China; 4 University of Chinese Academy of Sciences, Beijing 100049, China; 5 Department of Bioscience and Bioengineering, School of Chemistry and Biological Engineering, University of Science and Technology Beijing, 30 Xueyuan Road, Beijing 100083, China

**Keywords:** artificial small molecules, biological metal cluster mimics, coenzyme analogs, designer cofactors, non-natural nucleotides, non-natural amino acids, synthetic biology

## Abstract

Enzymes are essential catalysts for various chemical reactions in biological systems and often rely on metal ions or cofactors to stabilize their structure or perform functions. Improving enzyme performance has always been an important direction of protein engineering. In recent years, various artificial small molecules have been successfully used in enzyme engineering. The types of enzymatic reactions and metabolic pathways in cells can be expanded by the incorporation of these artificial small molecules either as cofactors or as building blocks of proteins and nucleic acids, which greatly promotes the development and application of biotechnology. In this review, we summarized research on artificial small molecules including biological metal cluster mimics, coenzyme analogs (mNADs), designer cofactors, non-natural nucleotides (XNAs), and non-natural amino acids (nnAAs), focusing on their design, synthesis, and applications as well as the current challenges in synthetic biology.

## 1. Introduction

Enzymes are extremely important catalysts and play a wide range of functions in the biological system. However, natural enzymes frequently cannot meet the catalytic requirements compatible with the metabolism of a chassis host in vivo, so engineering of the enzyme needs to be carried out on the cofactor specificity, substrate scope, and robustness [1]. With the development of synthetic biology, the engineering of enzymes is no longer limited to mutations of natural amino acids at different sites but increasingly involves the development of the low-cost, stable, and better-performing artificial small molecules. These artificial small molecules can serve as cofactors or building blocks of biomacromolecules, performing important biological functions, enhancing the performance of the enzymes, and expanding the types of biological metabolic pathways and biocatalytic reactions. A series of enzymes containing artificial small molecules have been obtained with improved activity, stability, stereoselectivity, and cofactor specificity. Metalloenzymes are a class of widely distributed enzymes that are frequently utilized for various challenging catalytic reactions. Important hydrogenases for biological hydrogen production, including [Ni-Fe] hydrogenase, [Fe-Fe] hydrogenase, and [Fe] hydrogenase [2], contain complex metal clusters and various cofactors. Researchers have made many efforts in metal center substitution, design, and incorporating artificial metal clusters as cofactors such as [Ni-Ru], [Ni-Mn], and NiRd [3,4]. The most widely used metalloprotein, hemoprotein, has successfully undergone the engineering of its cofactor heme, and designed artificial heme cofactors have been successfully used for cytochrome P450 enzymes [5]. Many redox enzymes require cofactors (coenzymes) to complete catalytic reactions with different cofactor preferences. To address problems such as an imbalance between supply and demand, a high production cost, and the low stability of cofactors in metabolic processes, non-natural coenzymes (mNADs) such as nicotinamide cytosine dinucleotide (NCD) and nicotinamide mononucleotide (NMN) have been biosynthesized [6,7,8]. Combined with random mutagenesis, semi-rational design, and rational design, the mutant enzymes specifically using mNADs as coenzymes have been realized [9,10,11]. Using designer cofactors, P450 enzymes have been engineered to catalyze novel reactions for non-natural substrates [12]. In addition, new components such as non-natural nucleotides (XNAs) and non-natural amino acids (nnAAs) are increasingly being used in semi-synthetic organism creation [13], improving enzyme properties and functions, and solving challenging biological problems [14].

Research on biological metal cluster mimics, coenzyme analogs, designer cofactors, and non-natural nucleotides and amino acids has made a lot of progress in recent years. Currently, the synthesis and engineering of artificial small molecules are becoming increasingly sophisticated, which not only promote the exploration and development of cellular metabolic pathways but also play an important role in synthetic biology and metabolism engineering. This review aims to summarize the design, synthesis, and application of artificial small molecules in synthetic biology and discuss current challenges and future directions in research.

## 2. Biological Metal Cluster Mimics

Some of the most challenging chemical reactions in nature, such as water splitting in photosynthesis and the reduction of N_2_ to NH_3_ in biological nitrogen fixation, are catalyzed by enzymes with metal cofactors under mild conditions without a high temperature or high pressure. The engineering of these enzymes is of great interest in both biofuel and biochemical production. Many researchers have produced artificial metalloenzymes with new functions by reusing the natural protein [15] or building entirely new artificial metalloproteins from scratch [16].

### 2.1. Metal Clusters of Artificial Hydrogenases

Due to the negative impact of non-renewable resources such as fossil fuels on the environment, the development of sustainable energy sources is particularly important. H_2_, as a clean alternative fuel, can greatly reduce atmospheric pollution and is expected to become a sustainable green energy carrier and storage method [17]. As important enzymes in biological hydrogen production, hydrogenases can reversibly convert H_2_ into protons and electrons [18]. According to their different metal ions in the active centers, hydrogenases are classified into [Ni-Fe] hydrogenases, [Fe-Fe] hydrogenases, and [Fe] hydrogenases [2]. The first two types of hydrogenases have different sensitivities to O_2_. [Ni-Fe] hydrogenase is reversibly inactivated by O_2_, while [Fe-Fe] hydrogenase is highly sensitive to O_2_ [19]. As a type of metalloenzyme, hydrogenases contain complex metal clusters and various auxiliary factors which have the potential to be engineered.

#### 2.1.1. Metal Clusters of Artificial [Ni-Fe] Hydrogenases

**Figure 1:**
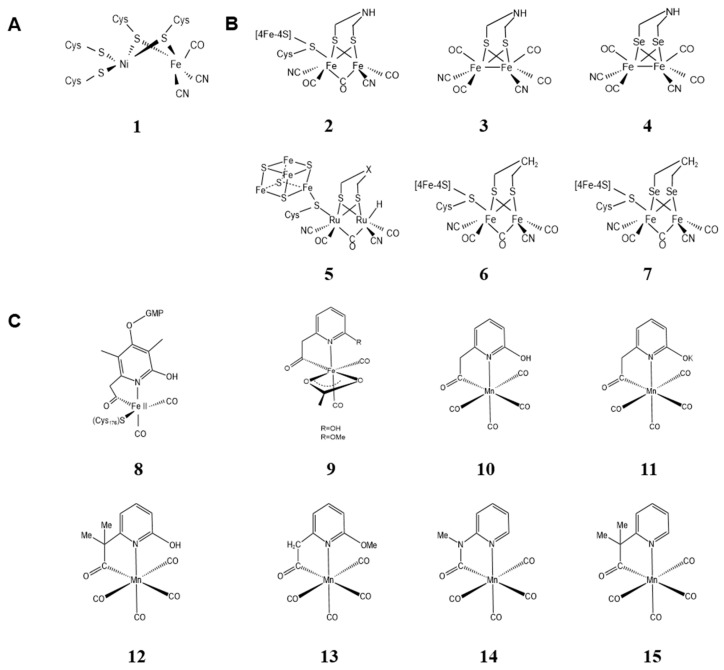
The metal clusters of native and artificial hydrogenases. (**A**) (**1**) Metal cluster structure of native [Ni-Fe] hydrogenases [19]; (**B**) metal cluster structure of [Fe-Fe] hydrogenases: (**2**) native metal cluster [19]; (**3**) ADT [35]; (**4**) ADSe [36]; (**5**) [Ru-Ru] hydrogenase [37]; (**6**) CrHydA1(pdt) [38]; (**7**) HydA1-PDSe [39]; (**C**) metal cluster structure of [Fe] hydrogenases: (**8**) native metal cluster [19]; (**9**) [47]; (**10**,**11**) [48]; (**12**–**15**) [49].

Since the first crystal structure of [Ni-Fe] hydrogenase (DG) was published by Volbeda et al. at 2.85 Å in 1995 [20], there have been increasing numbers of the structures and/or functions of [Ni-Fe] hydrogenases (Figure 1A). [Ni-Fe] hydrogenases rely on heterodimeric [Ni-Fe] cofactors to function. Because the synthesis of heterogenous bimetallic cofactors is difficult and their electronic structures are more complex, there is little research on directly modifying the metal co-factors of [Ni-Fe] hydrogenases or integrating artificially synthesized co-factors into [Ni-Fe] hydrogenases. Instead, the focus is mainly on simulating the active site structure of [Ni-Fe] hydrogenases and using the formed simulated complexes as catalysts for H_2_ production. For example, there are currently many heterobimetallic transition metal complexes—Ni-Fe [21,22,23,24], Ni-Ru [3,25,26], and Ni-Mn [4,27] model complexes—being used as catalysts.

In addition, researchers have attempted to achieve the [Ni-Fe] hydrogenase activity in other proteins. Slater et al. prepared nickel-substituted rubredoxin (NiRd), which has the same four-sulfur-coordinated environment as natural [Ni-Fe] hydrogenase around monovalent nickel [28,29]. The study found that the recombinant NiRd from *Desulfovibrio desulfuricans* ATCC 27,774 showed high H_2_ production activity in the solution [28]. Additionally, this NiRd protein had an advantage over natural [Ni-Fe] hydrogenases, since the NiRd protein is completely insensitive to the presence of O_2_ [29].

#### 2.1.2. Metal Clusters of Artificial [Fe-Fe] Hydrogenases

[Fe-Fe] hydrogenases have the highest efficiency in the process of proton reduction to H_2_, with a conversion frequency of up to 10^4^ s^−1^ [30]. Many researchers studied the biomimetic simulation of [Fe-Fe] hydrogenase metal clusters, mainly by simulating the two iron sites of the H cluster (Figure 1B), with azadithiolate (ADT) linking the two Fe atoms of [Fe-Fe] hydrogenases [31].

The biosynthesis of [2Fe] subclusters in vivo requires the involvement of the specific maturation proteins HydF, HydE, and HydG for hydrogenases. The role of HydF is to transfer the [2Fe] subcluster mimic to the carrier lipid hydrogenase for activation. *E. coli* lacks the three maturation proteins. When HydF is expressed heterogeneously in *E. coli*, the [2Fe] subcluster-deficient hydrogenase can be artificially matured to produce functional enzymes with [2Fe] subcluster mimics. Therefore, this artificial [2Fe]^adt^ maturation can be used to screen for [Fe-Fe] hydrogenases without the expression of the three maturation proteins simultaneously [32,33,34,35]. Furthermore, Esselborn et al. found that in the absence of the accessory protein HydF, a mimic synthesized by adding a CO group to the base of the H-cluster can be directly inserted into non-active apo-HYDA1 to form a fully active hydrogenase [35]. Kertess et al. synthesized fully active enzymes using another different method of metal substitution. They replaced the dithiolate ligand with Se to form ADSe and successfully integrated it into apo-HydA1 and apo-CpI in vitro. Compared with wild-type hydrogenases, the selenium-containing enzyme is more biased towards H_2_ production [36].

In most cases, even slight variations in co-factors can lead to a significant decrease in the catalytic activity of hydrogenases. Therefore, researchers have attempted to modify the metal co-factors of hydrogenases to gain a deeper understanding of their functional characteristics. They have made many efforts in the semi-synthetic method. An artificial [Ru-Ru] hydrogenase has been obtained by replacing two Fe atoms with the rare metal element ruthenium. It has the advantage of capturing key hydride intermediates [37]. Adamska-Venkatesh et al. used -CH_2_ to replace the -NH of the [2Fe] subcluster to form a biomimetic complex [Fe_2_(pdt)(CO)_4_(CN)_2_]^2−^ and then integrated it into the [Fe-Fe] hydrogenase of *Chlamydomonas reinhardtii* (CrHydA1). The artificial hydrogenase was found to be stabilized in a state similar to the oxidized state H_OX_ [38]. Sommer et al. also used the same analog synthesis method mentioned above to replace ADT with PDT, resulting in a low-activity enzyme with simplified redox behavior. They also obtained the HydA1-PDSe enzyme with the same redox behavior as HydA1-PDT [39].

Researchers have used various de novo-designed proteins to preserve [2Fe] metal cofactors, attempting to explore the behavior of [Fe-Fe] hydrogenase catalytic cofactors in a simpler environment than natural hydrogenases [40]. Some studies have emphasized the necessity of pre-organized Cys ligands in a rigid scaffold [41]. Jones et al. found that the peptide motif CXXC has a dual function of pre-organizing Cys ligands and providing water solubility for the complex [42]. Subsequently, they successfully applied this method to construct functional models by doping (μ-S-Cys)_2_Fe_2_(CO)_6_ into apo-cyt c with the CXXC motif. It was found that this artificial complex can release H_2_ through photocatalytic cycles of a ruthenium photosensitizer under mild conditions in aqueous media [43]. Subsequent studies on the metal cluster mimics mainly focused on designing more refined peptide structures due to the asymmetric substitution and functional splitting of the [2Fe] site in [Fe-Fe] hydrogenases.

#### 2.1.3. Metal Clusters of Artificial [Fe] Hydrogenases

[Fe] hydrogenases (Hmd) only exist in methanogenic archaea and contain a mononuclear iron center, with FeGP being its catalytic auxiliary factor. By denaturing the hydrogenase in the presence of 2-mercaptoethanol or acetic acid, the FeGP cofactor can be extracted. Reconstituting the cofactor with apo-[Fe] hydrogenase can rebuild active [Fe] hydrogenase [44,45]. Therefore, the strong extraction/reconstitution ability of this auxiliary factor is the basis for studying the characteristics of metal cofactors. It paves the way for constructing artificial hydrogenases containing non-natural auxiliary factors.

The Fe ion of [Fe] hydrogenase is coordinated by two cis CO ligands, a cysteine-derived thiol ligand and one bi-dentate 2-acetylpyridine-6-(N-alkyl) thiosemicarbazone ligand [19,46]. Since the structure of the FeGP metal cofactor of [Fe] hydrogenase was determined clearly, many Hmd synthetic models have been reported (Figure 1C). In 2015, Shima et al. synthesized two FeGP metal cofactor mimics and inserted them into recombinant apo-[Fe] hydrogenase in *E. coli*. The first active semi-synthetic [Fe] hydrogenase was successfully constructed, and it showed a higher turnover rate than most known synthetic catalysts but was found not to activate H_2_ [47]. 

Inspired by Mn-catalyzed hydrogenation reactions, Pan et al. incorporated an Mn(I) model into apo-Hmd and obtained an active [Mn] hydrogenase that can heterolytically cleave H_2_ at room temperature [48]. They then developed a series of Mn^I^ mimics of the active site of [Fe] hydrogenases [49]. Compared with semi-synthetic [Fe] hydrogenase, the catalytic activity of semi-synthetic [Mn] hydrogenase is higher. But it should be noted that [Mn] hydrogenase had a stronger forward reaction tendency [50]. Because Mn^I^ mimics are more stable than Fe^II^ mimics, the synthesis of catalytic [Mn] hydrogenase paved the way for structure–activity studies.

[Fe] hydrogenase contains only one metal, Fe, and can efficiently perform heterolytic cleavage and H^−^ transfer in low-H_2_-concentration aqueous media. Therefore, it can be used as an alternative or supplement to traditional hydrogenation catalysts. However, due to certain issues in enzyme preparation, substrate limitations, and functional activity improvement, the application of this enzyme and its artificial models in practical hydrogenation processes has not been widely explored yet.

### 2.2. Metal Clusters of Artificial Hemoprotein

**Figure 2:**
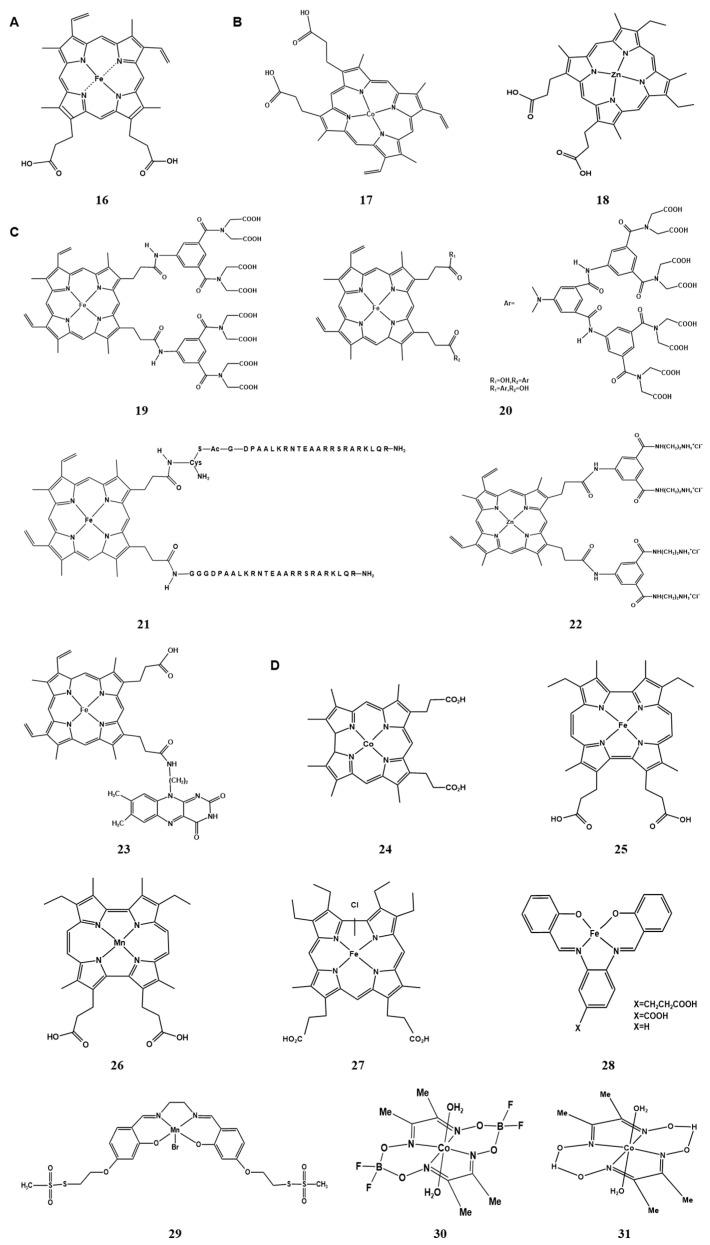
The metal clusters of native and artificial hemoprotein. (**A**) (**16**) heme (Fe-PIX) [51]; (**B**) metal substitution of heme: (**17**) CoPPIX [60,62]; (**18**) ZnPP [63]; (**C**) modify the propionic side chain: (**19**) double-winged cofactor [64,65]; (**20**) single-winged cofactor [64]; (**21**) heme(br)_2_ [66]; (**22**) [67]; (**23**) [68]; (**D**) non-natural porphyrin scaffolds: (**24**) corrin: Co(TDHC) [69]; (**25**) porphycene: [71,72,73]; (**26**) porphycene: MnPc [75,76]; (**27**) corrole: FeCor [77]; (**D**) non-porphyrin scaffolds: (**28**) [78]; (**29**) Mn(salen) [79]; (**30**,**31**) [80].

Hemoprotein is one of the most widely used metalloproteins and plays an important role in oxygen transport and storage, electron transfer, and catalysis in biological systems. Its cofactor is iron porphyrin (heme) (Fe-PIX) (Figure 2A) [51]. The simplest major cofactor in nature is heme b, also known as Fe protoporphyrin IX (FePPIX), which has three different peripheral substituents composed of four methyls, two vinyls, and two propionic side chains [52]. Through coordination, hydrogen bonding, hydrophobicity, and electrostatic interactions, heme b binds non-covalently to the heme pocket in proteins. And it is used as a cofactor in myoglobin, hemoglobin, horseradish peroxidase (HRP), cytochrome b5, and cytochrome P450. These proteins are converted to corresponding apolipoproteins after removing heme cofactors under acidic conditions, leaving a cavity as the heme coordination sphere [53]. The heme cofactor works as a reaction center to provide biochemical functions ranging from O_2_ or NO storage and transport to catalysis and electron transfer. These functions mainly come from the unique arrangement between the cofactor and protein matrix formed by the ferrous heme pocket.

Among these hemoproteins with heme b as a cofactor, myoglobin is a focus of many studies because it is a storage hemoglobin. However, the heme pocket of the myoglobin has been arranged only to stabilize the heme-bound dioxygen, so the structure is not suitable for the activation of small molecules such as H_2_O_2_ and O_2_ as well as for binding external substrates. Thus, converting myoglobin into an enzyme-like biocatalyst has important implications. Currently, there are three main methods for designing artificial heme cofactors: (i) the metal substitution of heme; (ii) the modification of peripheral functional groups of porphyrin ligands; and (iii) providing non-natural porphyrin/non-porphyrin cofactor skeletons [52,54].

#### 2.2.1. Metal Substitution of Heme in the Cofactor

Natural heme enzymes are capable of catalyzing C-H oxidation and halogenation reactions, and Fe-PIX proteins have also been shown to catalyze non-biological reactions, including the addition and insertion of carbene and nitroalkene [55]. However, due to the inherent reactivity of metal ions/cofactors themselves, these reactions are subject to certain limitations. For example, Fe-PIX proteins catalyze the insertion of carbene into strongly reactive N-H bonds and S-H bonds but do not catalyze the insertion into weakly reactive C-H bonds [55,56,57]. Dydio et al. discovered that a variant of CYP119 containing iridium-substituted iron could catalyze carbene insertion into C-H bonds with high enantioselectivity [58]. The method proposed by them for preparing artificial heme proteins containing non-biological metal porphyrins laid the foundation for generating artificial enzymes from the combination of a PIX-protein scaffold and non-natural metal cofactors [55]. In addition, Wolf et al. produced an artificial metal enzyme using ruthenium porphyrin IX recombinantly, and the results showed that RuMb was an effective N-H insertion catalyst. Compared with natural Mb, RuMb was a more active catalyst in carbene transfer reactions [59].

Compared with transition metals, cobalt is abundant and relatively inexpensive, making cobalt metal substitution important. Generally, *E. coli* can produce a new natural CoPPIX cofactor without genetic engineering, evolutionary adaptation, or auxiliary plasmids. Its efficiency in biosynthesis and integration into various heterologously expressed hemoproteins is similar to those of the natural FePPIX cofactor (Figure 2B) [60]. Sommer et al. used Co substitution to produce cobalt myoglobin, which can induce hydrogen production under mild aerobic conditions [61]. In addition, CoMb can also photo-catalyze the reduction of CO_2_ to CO in the presence of [Ru(bpy)_3_]^2+^, with the highest product selectivity among engineered enzymes [62]. Therefore, enzymes with non-natural or artificially introduced metal centers can generate new reactivities, catalyzing unexpected and novel reactions. For example, Shi et al. reported an artificial Mb constructed from zinc protoporphyrin, which exhibited new light-induced DNA cleavage activity [63].

#### 2.2.2. Modification of Peripheral Functional Groups of Porphyrin Ligands

Mb and HRP have the same cofactor, heme b, and both have two His residues at the proximal and distal ends [52]. However, the peroxidase activity of Mb is much lower than that of HRP, because (i) while H_2_O_2_ binds to heme in myoglobin, it is not properly activated to provide peroxidase activity, and (ii) there is no obvious substrate-binding domain in myoglobin [53]. The propionic side chain in myoglobin stabilizes and regulates the heme pocket through charge–charge interactions and/or hydrogen bonding with amino acid residues and to some extent regulates the function of Mb [51]. Various groups attached to the propionic acid side chain can serve as artificial substrate binding domains, helping organic substrates/proteins to enter the vicinity of ferrous heme in Mb, which is the key to the peroxidase activity of Mb. Asp residue can serve as an acidic-basic unit to activate H_2_O_2_, so the mutant H64D has an environment similar to HRP [64].

Therefore, based on the above method, Hayashi et al. introduced the H64D mutation and introduced an aromatic group at the end of the propionic acid side chain (Figure 2C). They used synthetic chemical strategies to connect the substrate binding domain “double-winged cofactor”. The peroxidase activity of the resulting artificial reconstituted Mb was significantly improved, with a k_cat_/K_m_ only three times lower than that of HRP. However, it was found that this modification caused the k_cat_ value of the mutant Mb to be much lower than that of the mutant containing native heme [64,65]. Therefore, in subsequent studies, they incorporated a “single-winged cofactor” into apo-H64D Mb to reconstitute the protein. As a result, the formed reconstituted Mb was found to have a larger k_cat_ value, and its peroxidase activity was equivalent to that of HRP. This indicates that the significance of an unmodified propionic acid side chain is to stabilize the orientation of the heme in the heme pocket and form hydrogen bonds [64]. In addition, Sakamoto et al. introduced another molecule into the propionic acid side chain, a peptide heme (br)_2_, as an artificial DNA binding site and incorporated it into apo-Mb to obtain Mb(br)_2_, which also enhanced peroxidase activity [66]. 

By modifying the propionic acid side chain of heme b, hemoprotein can be transformed into a protein with new functions. For example, to create a protein that triggers light-induced electron transfer, researchers used modified zinc protoporphyrin recombinantly and introduced four ammonium groups at the end of the two propionic acid side chains [67]. Some studies have introduced a flavin into the propionic acid side chains of Mb, which gives the recombinant Mb an electron transfer mechanism similar to that of cytochrome P450. This artificial Mb can activate dioxygen, and it has been successfully converted to an oxygen-activating hemoprotein [68]. 

#### 2.2.3. Providing Non-Natural Porphyrin/Non-Porphyrin Cofactor Scaffolds

Relative to the natural porphyrin scaffold, the porphyrin-like scaffold has unique characteristics and different structures (Figure 2D). Therefore, non-natural porphyrin-like scaffold metal complexes have significantly different reactive activities compared to heme [54]. Researchers have designed and synthesized various artificial porphyrin analog compounds to change the redox and reactive activity of the metal ion in the porphyrin nucleus in order to achieve the functional modification and improvement of hemoproteins [52].

Corrin is a monoanionic porphyrinoid ligand that lacks one of the four central carbons in the porphyrin framework. Hayashi et al. designed and prepared tetradehydrocorrin and incorporated it into the heme pocket of apo-Mb to form an artificial recombinant Co (TDHC). It can serve as a simple model for the active site of a complex cobalamin-dependent methyltransferase [69].

Porphycene is an isomer of porphyrin and a dianionic porphyrinoid used to construct artificial metal proteins. It has lower symmetry than porphyrin [54,70]. Some physical and chemical properties of porphycene are significantly different from those of porphyrin, and it has been found that hemoprotein recombined with metal porphycene exhibits unique properties.

Hayashi et al. designed and produced a novel myoglobin FePc reconstructed with iron porphycene and found that it had a significantly higher oxygen binding affinity and peroxidase activity than natural Mb [71,72,73]. In 2007, they also incorporated iron porphycene into the heme pocket of HRP and found that this protein had higher reactivity towards the oxidation of thioanisole [74]. Natural myoglobin does not have hydroxylation activity, but some studies have prepared an Mn porphycene myoglobin (rMb (MnPc)) and incorporated it into apo-Mb, finding that it significantly enhanced the catalytic activity of myoglobin towards the C(sp3)-H hydroxylation of substrates [75,76].

Corrole is a trianionic porphyrin ligand that lacks a carbon atom between two of the four pyrrole units compared to the porphyrin scaffold [52]. Matsuo et al. designed and synthesized an iron corrole complex (FeCor) and incorporated it into apo-Mb and apo-HRP. It was found that the recombinant myoglobin exhibited significantly enhanced peroxidase activity [77].

Non-porphyrin cofactor ligands have also been widely studied as cofactors for artificial metalloenzymes (Figure 2D), leading to the development of some recombinant proteins with new functions. Ueno et al. prepared a hybrid metalloprotein using apo-Mb and a Schiff-base complex, which promoted the rapid consumption of NADH and O_2_ in heme oxygenase [78]. Carey et al. used a new dual-covalent anchoring binding strategy to incorporate Mn(salen) into apo-Mb. It was found that this method significantly improved the enantioselectivity of the semisynthetic enzyme and increased the rate of the sulfoxidation of thioanisole [79]. Bacchi et al. created an artificial recombinant protein with hydrogenation enzyme properties by incorporating two cobaloximes into apo-SwMb, which catalyzed H_2_ evolution at low overpotentials [80].

### 2.3. Metal Clusters of the Artificial Photosynthesis System

In nature, solar energy is utilized and stored through photosynthesis including both light and dark reactions in two photosystems: Photosystem I and II (PSI and PSII). Green plants, algae, and cyanobacteria convert solar energy into protons, electrons, and O_2_ through water oxidation, providing protons and electrons for the second half of the reaction (H_2_ production) of water splitting, and H_2_ can be used as a new energy source [81]. The water oxidation process is catalyzed by PSII.

2H_2_O → O_2_ + 4H^+^ + 4e^−^

PSII is a large protein complex composed of more than 20 subunits, with a molecular weight of approximately 350 kDa [82,83]. Its essential elements are (i) a strongly oxidizing multichlorophyll complex termed P680; (ii) a redox-active tyrosine termed Y_Z_; (iii) a bound plastoquinone electron acceptor; and (iv) a metalloprotein containing a manganese, calcium cluster called the oxygen-evolving center (OEC) [84,85]. In 2011, Umena et al. reported the crystal structure of PSII at a resolution of 1.9 Å and identified the metal cluster in the OEC as Mn_4_O_5_-Ca(H_2_O)_4_ [83]. The OEC is capable of water oxidation at a low overpotential (≈0.18 V) and a high rate (≈500 s^−1^) [85]. So, the rate of O_2_ production by the oxygen-evolving complex (OEC) is comparatively high.

Currently, studies are mainly focused on synthesizing relatively simple metal analogs that combine catalytic activity with chemical stability to achieve the catalytic efficiency of the OEC. As a result, many molecular complexes have been developed to catalyze water oxidation. Early research on water oxidation catalysts focused mainly on complexes based on Ru [86,87,88] and Ir [89,90,91], while current research has shifted its focus to non-noble metal catalysts based on abundant metals on Earth such as Fe [92,93], Co [94,95], Ni [96,97,98], and Cu [99,100,101]. These studies have deepened our understanding of the mechanism of water oxidation and resulted in a series of catalytic agents with activity in water oxidation. These artificially synthesized water oxidation catalysts can provide possibilities for sustainable artificial photosynthesis.

The complexity of PSII makes its direct application in fuel production impractical, but the de novo design of proteins provides a new approach to artificial photosynthesis. Conlan et al. used a modified form of bacterioferritin (BFR) from *E. coli* as a protein scaffold to propose an artificial PSII model [102]. The reconstruction of the photosynthetic reaction center offers a way to directly redesign photosynthesis for human needs. Researchers have developed a single-reaction-center photosynthetic system that supports water oxidation and proton reduction. This model system achieves long-lived, light-driven charge separation and contains many elements from natural photosynthetic reaction centers [103]. In addition, researchers have used the template Df2t homodimer protein structure to design three different four-helix bundle proteins, P0, P1, and P2, de novo, each containing one, two, or three dinuclear Mn centers. These artificial manganese proteins are capable of transferring electrons to bacterial reaction centers, providing an opportunity to study the oxidation-reduction properties of the dinuclear manganese cofactor [104].

## 3. Coenzyme Analogs

Oxidoreductases are the largest group of enzymes reported to date, and their catalytic reactions typically require coenzymes to transfer electrons, hydrogen, oxygen, or other small-molecule intermediates [105]. Typical coenzymes include nicotinamide adenine dinucleotide (NAD), nicotinamide adenine dinucleotide phosphate (NADP), ubiquinone (CoQ), and flavin mononucleotide (FMN/FAD), among which NAD, dependent for 80% of oxidoreductases, is the most popular. NAD and its reduced form NADH are essential electron carriers for many redox reactions and substrates for some biological reactions, playing important roles in cellular metabolism [106,107]. However, natural coenzymes like NAD are unstable, expensive to use in vitro, and difficult to control in vivo [108], which limits their application in large-scale synthesis and intrigues researchers to develop non-natural coenzymes. Non-natural coenzymes (mNADs) have industrial value in reducing feedstock costs because mNADs are generally simpler to synthesize and have greater stability than natural coenzymes [109], allowing access to new chemicals with altered redox potentials [110] and enabling the specific delivery of electrons [9]. At the same time, mNADs are valuable molecular tools for detecting, monitoring, structurally studying, and regulating the activity of NAD-related enzymes and biological processes. A biocompatible emissive mNAD has been designed to realize the real-time visualization of cofactor-dependent processes through fluorescence spectroscopy [111].

**Figure 3:**
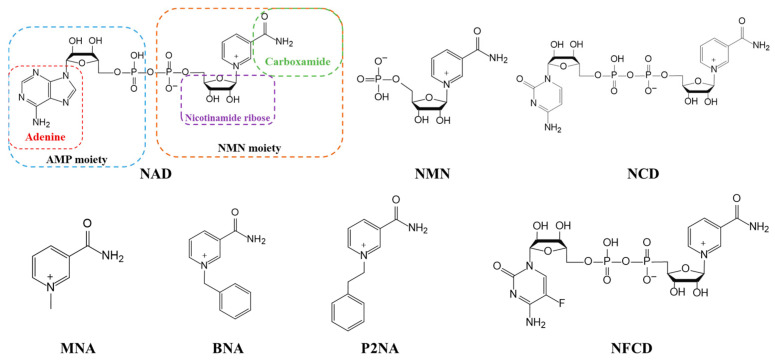
Chemical structures of NAD and mNADs. Full names of the mNADs: MNA, 1-methyl-1,4-dihydropyridine-3-carboxamide; BNA, 1-benzyl-1,4-dihydronicotinamide; P2NA, 1-phenethyl-1,4-dihydropyridine-3-carboxamide; NMN, Nicotinamide mononucleotide; NCD, Nicotinamide cytosine dinucleotide; NFCD, Nicotinamide flucytosine dinucleotide.

The structure of natural coenzymes is divided into the nicotinamide mononucleotide (NMN) moiety responsible for transferring hydrogen and electrons and the adenosine phosphate (AMP) moiety responsible for anchoring the coenzyme and enzyme interaction. Some mNADs were obtained by modifying or replacing the carboxamide [112], the adenine base [113,114,115], and the nicotinamide ribose [116] of NAD with alternative functional groups, as shown in Figure 3. Since the redox chemistry of NAD occurs in the NMN moiety, the synthesis of mNADs mainly involves modifying the AMP moiety of natural coenzymes [117] and can be classified into two categories. One is semi-synthetic biomimetic coenzymes that are similar to natural nicotinamide coenzymes in structure, usually in truncated forms of natural coenzymes (such as NMN) or only modifying and replacing some groups of natural nicotinamide coenzymes [9,10]. The other category is fully synthetic biomimetic coenzymes [118], which are typically small in size (e.g., BNA) and retain only the niacinamide group responsible for electron transfer.

**Figure 4:**
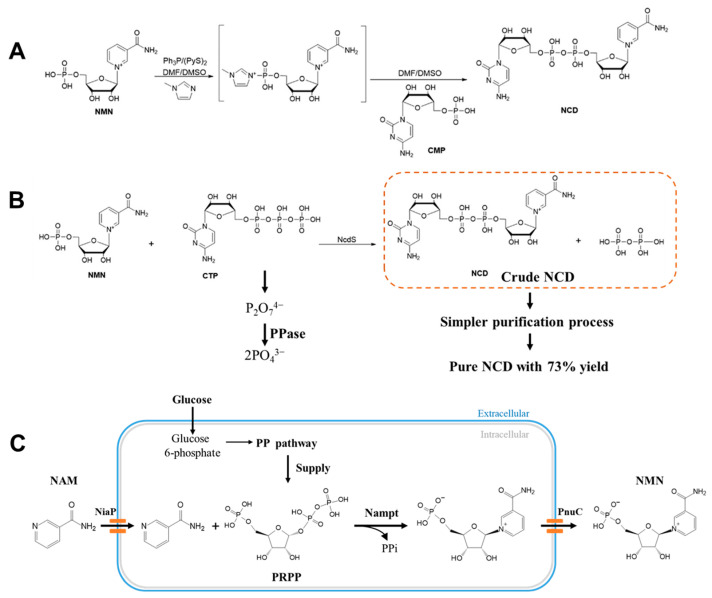
Reactions related to mNADs synthesis. (**A**) Chemical synthesis of NCD; (**B**) Biosynthesis of NCD using CTP and NMN; (**C**) Biosynthesis of NMN using glucose and NAM.

A series of biologically active NAD analogs have been reported, such as non-natural coenzyme nicotinamide cytosine dinucleotide (NCD), with only one base difference from NAD [119]. Most of these mNADs were chemically or chemoenzymatically synthesized, including N^TZ^AD containing thiazolo [4, 3-d] pyrimidine moiety [111] and 4′-thioribose NAD [120]. It is worth noting that, except for MNA, BNA, P2NA, and other stable compounds that could be easily synthesized by treating nicotinamide with alkyl halides [121], most of the chemical synthesis methods were complicated with low product yields. For example, NCD prepared by the chemical synthesis of NMN and CMP (Figure 4A) upon tedious post-processing could yield only about 100 mg per batch [6]. To facilitate the mNADs-linked redox chemistry in vivo, it is essential to achieve the biosynthesis of mNADs. In 2022, Zhao and his colleagues developed a simple and rapid method (Figure 4B) for achieving the gram-scale biocatalytic preparation of NCD [6]. First, they generated an NCD synthetase (NcdS-1) by reprogramming the substrate binding pocket of nicotinic acid mononucleotide (NaMN) adenylyltransferase, enabling it to be favorable to cytidine triphosphate (CTP) and NMN, respectively, rather than their conventional substrates adenine triphosphate (ATP) and NaMN [122,123]. With the help of Ncds-1, the efficient catalysis of NMN and CTP was achieved. Subsequently, the inorganic pyrophosphatase (PPase) from *Escherichia coli* was used to optimize the reaction composition and achieved a near-quantitative conversion of substrates. By centrifugation, ultrafiltration, concentration, precipitation, and ion exchange chromatography, they obtained a 4.19 g NCD product with 96% absolute purity from a production scale of 300 mL and 30 mM NCD, ultimately.

In addition, the synthetic precursor of NCD, NMN, has also been biosynthesized in *E. coli*. Yu et al. [7] adopted synthetic biology strategies to design strains with improved performance, including screening for exogenous Nampt enzymes, enhancing the availability of precursor phosphoribosyl pyrophosphate (PRPP) and ATP, and exploring homologs of natural nicotinamide (NAM) transporters. Combined with the process optimization of whole-cell biocatalyst reactions, a simple biotransformation process was developed for the synthesis of NMN from an inexpensive substrate NAM and glucose, and an NMN titer of 496.2 mg L^−1^ was obtained. During the same period, a study reported the synthesis of the highest yield of NMN to date, which produced 6.79 g L^−1^ NMN extracellularly from glucose and NAM by an engineered *E. coli* BL21(DE3) strain with a plasmid-based protein expression system (Figure 4C) [8]. They identified two active functional transporters (NiaP and PnuC) and a highly active key enzyme (Nampt) that allowed for NAM uptake, the efficient conversion of PRPP (provided by glucose) and NAM to NMN, as well as extracellular NMN excretion. The enhancement of the PRPP biosynthetic pathway and the optimization of individual gene expression resulted in much higher NMN production than previously reported. The biosynthesis methods described above are expected to be used for the industrial production of low-cost, high-quality mNADs by making use of microorganisms.

Most wild-type enzymes have poor utilization of mNADs, that is, they still prefer NAD. However, many flavor enzymes, such as enoate reductases, nitroreductases, and para-hydroxybenzoate hydroxylase, exhibit promising activity [9,124,125]. To expand the regulatory capacity of redox metabolism, efforts have been made to create mNADs-dependent oxidoreductases, including malic enzyme [119,126], phosphite dehydrogenase [115,127], formate dehydrogenase [128], glucose dehydrogenase [9], d-lactate dehydrogenase [113,119], methanol dehydrogenase [129], formaldehyde dehydrogenase [11], and so on. In general, the coenzyme preference modification of oxidoreductases can be achieved by classical protein engineering methods: random mutagenesis, semi-rational design, and rational design. Since most NAD(P)-dependent oxidoreductases have a highly conserved coenzyme-binding Rossmann fold sequence [130], semi-rational design and rational design are more widely used in oxidoreductase coenzyme engineering. Sieber et al. [116] carried out a rational design on glucose dehydrogenase (*Ss*GDH) derived from *Sulfolobus solfataricus* and screened a double-mutant Ile192Thr/Val306Ile through the site-specific saturation mutagenesis of nine amino acid residues near the coenzyme pocket. The mutant showed a 10-fold increase in enzymatic activity towards the non-natural coenzyme P2NA compared with the wild-type enzyme and was successfully applied in 2-methylbutanal production. Li et al. [9] achieved the maximum specific conversion (10^7^-fold) of glucose dehydrogenase (*Bs*GDH) from *Bacillus subtilis* to a non-natural coenzyme employing computer-aided design. The engineered mutant strictly relied on NMN and was successfully used for the enzymatic reaction in vitro. Similarly, Huang et al. successfully improved the activity of *Tm*6PGDH to NMN by utilizing high-throughput screening methods. The optimal mutant showed a 50-fold higher catalytic efficiency towards NMN than the wild-type enzyme, whose activity towards NMN was comparable to that of the wild-type enzyme towards the natural coenzyme NADP [10], and this could be used for in vitro synthetic biology.

**Figure 5:**
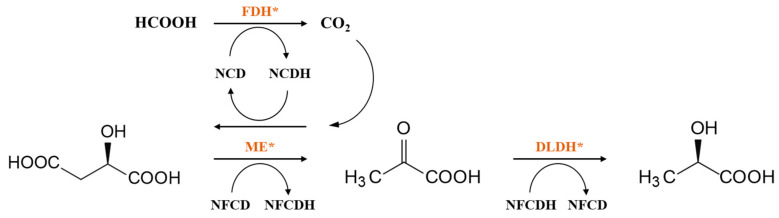
Non-natural coenzyme (mNADs) orthogonal system of NCD and NFCD with mutated formate dehydrogenase (FDH*), malic enzyme (ME*), and d-lactate dehydrogenase (DLDH*).

The research team led by Professor Zongbao Kent Zhao has made significant contributions to the application of mNADs in biological orthogonal systems. They used a semi-rational approach to evolve the phosphite dehydrogenase (Pdh) from *Ralstonia* sp. strain 4506 to obtain a mutant I151R/P176R/M207A that could utilize NCD as a coenzyme. The enzymatic activity of this mutant towards NCD and that of the wild-type enzyme towards NAD were found to be of the same order of magnitude. The team then summarized the general rules for modifying dehydrogenase preferences through the crystal structure and mechanism analysis. The basic principle is to shrink the volume of the coenzyme binding pocket by introducing residues with large side chains, which are unfavorable for NAD binding, and to achieve a preference for coenzymes with a reduced size [115]. It is of great significance for constructing engineered enzymes to utilize mNADs efficiently. Using the same method, the team obtained various NCD-preferred oxidoreductase mutants and constructed corresponding orthogonal redox catalytic systems and phosphite-driven organic acid synthesis systems. By coupling the engineered formate dehydrogenase (FDH*) [128] with malic enzyme (ME*) and _D_-lactate dehydrogenase (DLDH*) [119], they constructed a formate-driven, mNADs-mediated malate biosynthesis and oxidative decarboxylation system in microbial cells (Figure 5). As a result, NCD has become an artificial coenzyme successfully biosynthesized for use in intracellular orthogonal redox reactions. This research is of significant reference value for the artificial design of metabolic pathways and the selective regulation of cellular material energy metabolism.

**Figure 6:**
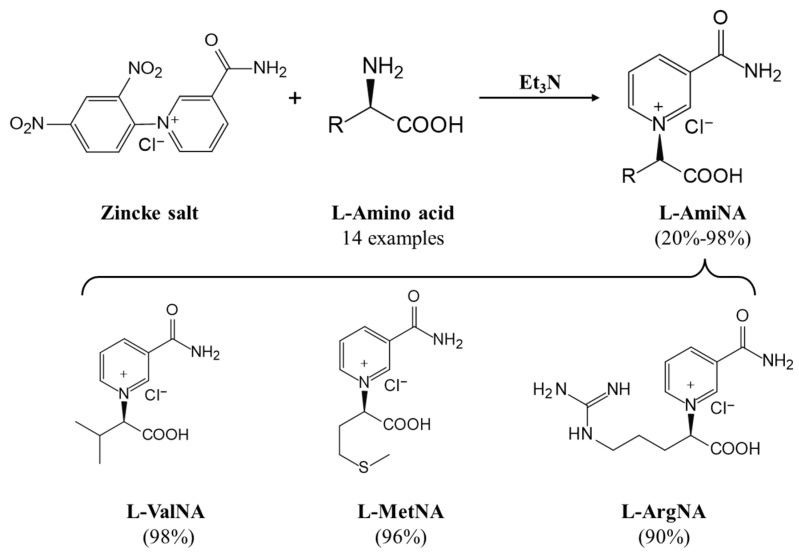
Synthesis of proteogenic amino acid-based mNADs.

It is worth mentioning that to further increase the structural diversity and biocompatibility of mNADs, Zhao’s team also synthesized 14 proteogenic amino acids-based NAD analogs through the Zincke reaction and characterized their physicochemical properties such as spectroscopy and redox potential [131]. Figure 6 showed mNADs with an isolation yield of 90% or above. Several representative mNADs were tested with the bifunctional fatty acid hydroxylase P450 BM3-R966D/W1046S [132], which was known to have relaxed coenzyme specificity, to explore whether l-AmiNAs could serve as oxidoreductase coenzymes. The preliminary screening results indicated that a few reduced analogs (including l-MetNAH) could effectively promote the fatty acid hydroxylation of the mutant cytochrome P450 enzyme as a coenzyme. However, the role of l-AmiNAHs as a redox enzyme coenzyme was still significantly reduced compared to NADH. Future consideration could be given to designing coenzyme binding pockets of redox enzymes to favor l-AmiNAHs.

In summary, research on the non-natural coenzymes preference of oxidoreductases is still a relatively new field, and the construction of an efficient non-natural coenzymes regeneration system is also in the primary stage. In addition to engineering enzymes to favor mNADs, designing other specific and efficient cofactors for specific enzymes is also an effective approach. In the next section, we will discuss the design of cofactors in detail by taking P450 enzymes as an example.

## 4. Designer Cofactors

Cytochrome P450 enzymes (CYPs or P450s), catalyzing the monooxygenation of various substrates, including aliphatic and aromatic compounds, alkenes, and heteroatoms, are promising versatile oxidative biocatalysts [133,134,135]. Despite their impressive ability to oxidize inert C-H bonds in a regio- and stereoselective manner, the preparative-scale applications of P450s in vitro are limited due to their dependence on the coenzyme NAD(P)H and the complex electron transport system [136]. Additionally, intrinsic drawbacks such as the narrow scope of useful nonnative substrates, poor stability, and low catalytic rates also limit the practical use of P450s. Protein engineering may help to overcome some of these issues; on the other hand, designer cofactors have been developed to create new catalytic capabilities of P450s and facilitate their further industrial utilization as biocatalysts in vitro [12].

Designer cofactors can be classified into several categories, including substrate anchoring groups, decoy molecules, and dual-functional small molecules (DFSMs). Substrate anchoring groups improve the substrate scope and reaction selectivity by engineering the substrate, using the concept of docking and protecting groups in bio-hydroxylation [137]. Decoy molecules with similar structures to the native substrate of P450s, particularly the moieties that are responsible for substrate binding, are used to reshape the active site of the enzymes to accommodate another non-native substrate [138,139]. On the other hand, DFSMs not only reform the active site like decoy molecules but also directly participate in the catalytic process, creating new catalytic capabilities towards non-native substrates [140]. 

**Figure 7:**
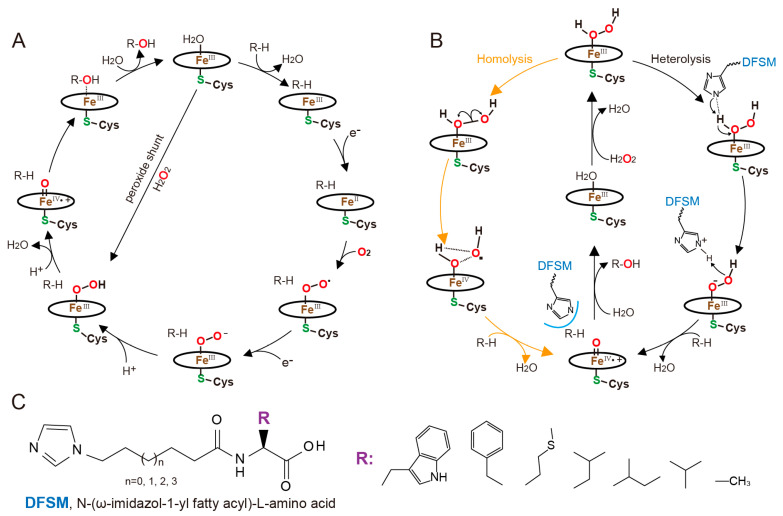
The catalytic cycle of cytochrome P450 monooxygenase and the peroxide-shunt pathway (**A**) and P450BM3-H_2_O_2_ system assisted by the DFSM (**B**) [143], and the representative structures of the dual-functional small molecules (DFSMs) (**C**) [142].

As is well known, there are two pathways for activating P450s, namely, the NAD(P)H-dependent and the peroxide shunt pathway (Figure 7A) [141]. Most native P450s adopt the NAD(P)H-dependent pathway and exhibit poor reactivity in the presence of H_2_O_2_. Therefore, shifting P450s from the NAD(P)H-dependent pathway to the H_2_O_2_-dependent pathway could be an attractive approach for their practical utilization. Recently, with the assistance of designer cofactor DFSMs, a unique strategy for an H_2_O_2_-driven P450BM3 system was developed by Cong and coworkers [142]. The DFSM comprises three parts, an acyl amino acid group responsible for binding to the enzyme as an anchoring group, an imidazolyl group serving as a general acid-base catalyst in the activation of H_2_O_2_, and a short fatty acid that connects them (Figure 7C) [142]. Computational investigations reveal that H_2_O_2_ activation by P450BM3 is highly dependent on the DFSM. In the absence of DFSM, the enzyme prefers homolytic O-O cleavage to form compound I (Cpd I), while in the presence of the DFSM, a proton channel formed between the imidazolyl group of the DFSM and the proximal H of H_2_O_2_, enabling a heterolytic O-O cleavage and Cpd I formation that is greatly favored over the homolysis mechanism (Figure 7B) [143].

This DFSM-facilitated P450-H_2_O_2_ system has been developed into versatile biocatalysts for many non-native substrates, showing the best peroxygenase activity for the epoxidation of styrene, the sulfoxidation of thioanisole, and the hydroxylation of ethylbenzene among the previously reported P450-H_2_O_2_ systems [142], as well as the selective hydroxylation of naphthalene [144]. Moreover, this H_2_O_2_-driven P450BM3 system can hydroxylate small alkanes with high regioselectivity, and its turnover number (TON) is comparable to that of the fungal H_2_O_2_-dependent natural alkane hydroxylase AaeUPO [145,146]. The product formation rates are also similar to or better than those of evolved/engineered NADPH-dependent P450 systems [146,147,148]. The regioselective *O*-demethylation of various aromatic ethers has been achieved by this DFSM-facilitated peroxygenase system with several simple mutants in the enzyme [149]. In addition to these high regioselective reactions, this system has also been developed into a highly enantioselective system, such as the epoxidation of styrene and its derivatives *o*-, *m*-, *p*-chlorostyrenes, and fluorostyrenes, with the (*R*)-enantiomeric excess (e.e.) of the products reaching up to 99% [150]. An even more exciting advancement in this design cofactor system is that the DFSM-facilitated system enables access to over half of all possible hydroxylated products from each given alkylbenzenes substrate, with excellent regioselectivity (up to 99%) and enantioselectivity (up to 99% e.e.) and high total TON (up to 80963) [151]. These results indicate that the synergistic use of an exogenous DFSM and protein engineering constitutes an efficient strategy for controlling the regio- and enantioselectivity of P450BM3 for non-native substrates.

In addition to its peroxygenation activity, this DFSM-facilitated P450-H_2_O_2_ system displays peroxidase activity and is reinforced towards various classic one-electron oxidation substrates through the combination of site-directed mutations on redox-sensitive residues [152]. It is worth noting that this modified P450 peroxidase system can also catalyze similar reactions by using nitrite as a nitrating agent. The nitration of multiple phenol and aniline compounds results in moderate-to-high total TONs of *ortho*- and *para*-nitration products. Furthermore, besides the direct aromatic nitration caused by P450 variants through using nitrite as a nitrating agent, this DFSM-facilitated P450 peroxidase system can also catalyze the nitration of the vinyl group of styrene and its derivatives [153].

In general, the designer cofactor DFSM is successful in shifting the NADH-dependent P450BM3 into its peroxygenase or peroxidase modes and simultaneously expanding the scope of substrates, which enriches the toolbox for developing practical P450 biocatalysts for the synthesis of commodity chemicals [154]. However, it is worth noting that there are still some drawbacks that hinder its further industrial use, such as the oxidative damage of P450 caused by the large amounts of H_2_O_2_, uncertainty about applying this strategy to other P450s, and the increased cost associated with introducing a large excess of DFSMs. Nevertheless, researchers are actively working on these limitations. Recent efforts by Cong and coworkers have aimed to address these limitations by engineering hydrogen peroxide tunnels in P450 monooxygenases, enabling peroxygenase activity and increasing the H_2_O_2_-driven activities of two native NADH-dependent P450 enzymes by >183-fold and >15-fold, respectively [155]. Moreover, the amount of H_2_O_2_ required for the DFSM-facilitated P450BM3 peroxygenase to obtain the desired product has been reduced by 95–97.5% (with an approximately 95% coupling efficiency) [155]. To further reduce the working concentration of DFSMs, the structure-guided optimization of DFSMs has been conducted, resulting in the identification of some unnatural amino acids with better performances [16]. This work has greatly enriched the DFSM toolbox for activating the peroxide-shunt pathway of P450s and could provide customized DFSM solutions for specific substrates and reactions.

## 5. XNAs and nnAAs

Besides the cofactors in proteins, various artificial small molecules have been developed for use as building blocks in biomacromolecules. With the development of synthetic biology, it is possible to incorporate non-natural nucleotides (XNAs) and non-natural amino acids (nnAAs) into genes and proteins through genetic manipulation. Due to their higher stability, activity, and excellent anti-degradation ability, some XNAs have been used in quantitative PCR (qPCR) methodology [156] and semi-synthetic organism creation. Similarly, more than 200 nnAAs have been incorporated into prokaryotes and eukaryotes [14] and have been used in a variety of explorations, including protein labeling [157], biomolecular targeting by reacting with fluorescent probes [158,159,160], protein interaction analysis [161], real-time tracking and in vivo imaging [162], biological containment system construction [163], the preparation of new biological materials [164], lanthipeptides bioengineering [165], and generating new biocatalysts. From this perspective, the incorporation of XNAs and nnAAs expanded the genetic code and central dogma, providing new genetic engineering tools for producing proteins with higher activity, better performance, and wider applications [166]. In this section, we will summarize the design of XNAs and the main methods of nnAAs incorporation, focusing on the applications of nnAAs in synthetic biology.

### 5.1. XNA Engineering

**Figure 8:**
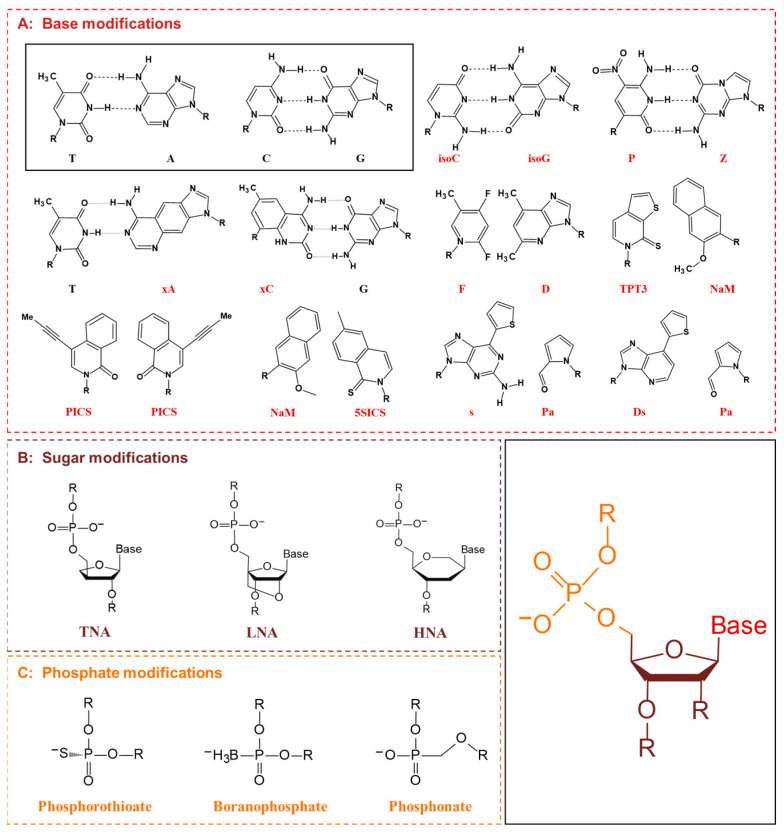
Chemical structures of XNAs mentioned in the review. (**A**) XNA with base modifications; (**B**) XNA with sugar modifications; (**C**) XNA with phosphate modifications.

Nucleotides consist of bases, five-carbon sugars (ribose or deoxyribose), and phosphate groups, all of which can be modified (Figure 8). Base modifications can change the base pairing characteristics and expand the information coding ability of nucleic acids. There are two main strategies for introducing the third base pairs apart from A-T and G-C, called UBPs, into DNA and RNA. The first aims to maintain the Watson–Crick-like hydrogen-bond network between the bases, and the second uses hydrophobic groups to mimic the shape and polarity of the natural bases. The pioneering work of the former came from the Benner laboratory, whose earliest design of isoG-isoC was confirmed to be recognized by DNA and RNA polymerase. However, under the condition of DNA synthesis, iso-C may slowly hydrolyze to U, and the iso-G may exist in the form of tautomerism complementary to U to some extent [167]. To solve these problems, Benner and his team redesigned a new base pair Z:P [168], which could be easily accepted by DNA polymerase, and the retention rate of the non-natural base pair was 97.5% per cycle of PCR amplification [169]. In contrast to the reassignment of Watson–Crick hydrogen bonds, Kool et al. synthesized the first hydrophobic base pairs F:D, which were shape-like analogs of natural bases for achieving specific pairing through hydrophobicity. They also studied four benzopyrimidine C-nucleosides analogs (xA, xC, xG, xT), and two of them (xA and xC) could be replicated by the polymerase in *E. coli* and produced correct messages [170]. However, it was not clear to what extent the incorporation efficiency was related to the shape complementarity. The idea of hydrophobic base pairing was further developed by Romesberg’s group, who first developed self-complementary pairs of propynyl-isocarbostyril (PICS) to overcome the requirement of shape complementarities [171]. Subsequently, Romesberg continued to optimize the hydrophobic base pair system, and the resultant 5STIC:NaM pair approached the efficiency of the natural base pair at every step of the replication [172]. More impressive, Hirao et al. designed a Pa:Ds base pair that could be efficiently amplified by PCR. Based on this, another fluorescent UBP was introduced into a selected site of RNA, and site-specific fluorescent probe tools based on s:Pa and Pa:Ds were established [173], which provided convenience for studying the local structure and intermolecular interaction of RNA. In recent years, researchers have successfully incorporated the hydrophobic base pair TPT3-NaM into *E. coli*. In this semi-synthetic organism, DNA containing these two UBPs was normally transcribed in vivo, resulting in the site-specific incorporation of natural and non-natural amino acids in green fluorescent protein. More importantly, semi-synthetic organisms could grow robustly and maintain the modified genetic material stably [13].

Besides bases, modifications of sugar rings and phosphate were also reported. The phosphates of threonine nucleic acid (TNA) are attached to oxygen on the 3′ and 2′ sites of furan sugars, and there is no methylene between the sugar ring and the oxygen atom on the phosphate [174]. TNA could be incorporated into DNA templates by Therminator DNA polymerase [175]. Similarly, locked nucleic acid (LNA) and hexitol nucleic acid (HNA) could also be introduced by different polymerases, such as KOD DNA polymerase, T7 RNA polymerase [176], or Vent (exo-) DNA polymerase [177]. For phosphates, phosphorothioate was the most common phosphodiester analog; it had mRNA activity and could be recognized by ribosomes [178]. Boranophosphates and phosphonate were also accepted by polymerases [179]. The latter contained additional methylene between the 5′ oxygen and phosphorus atoms and was highly resistant to nuclease degradation. It was reported that adenine phosphonate and cytosine phosphonate derivatives were favored by polymerase [180]. In conclusion, by incorporating XNAs into DNA and RNA, it is possible to improve the expression level and efficiency of the inserted genes due to the stability and resistance to the degradation of XNAs [181].

### 5.2. nnAAs Incorporation

Protein chemical modifications are important tools for elucidating and engineering biological functions, but their applications are limited to 20 natural amino acids. Non-natural amino acids (nnAAs), which can greatly expand protein engineering, are also of great interest. It would be beneficial for the characterization of the protein structure, protein interaction, and protein dynamics by introducing some nnAAs with functional groups. People have developed a variety of methods for incorporating nnAAs in vivo, of which residue-specific incorporation (RSI) and site-specific incorporation (SSI) are the most commonly used. RSI usually uses autotrophic systems for expression hosts to achieve the global substitution of specific natural amino acids, relying on the promiscuity of natural translation mechanisms. This method is simple and convenient and does not require genetic manipulation. However, due to the incorporation of nnAAs at multiple sites, the physicochemical properties of the obtained proteins are often greatly changed [182]. In contrast, SSI causes less disturbance to proteins, but it is extremely challenging to incorporate multiple different nnAAs at the same time, as it requires different genetic modifications.

**Figure 9:**
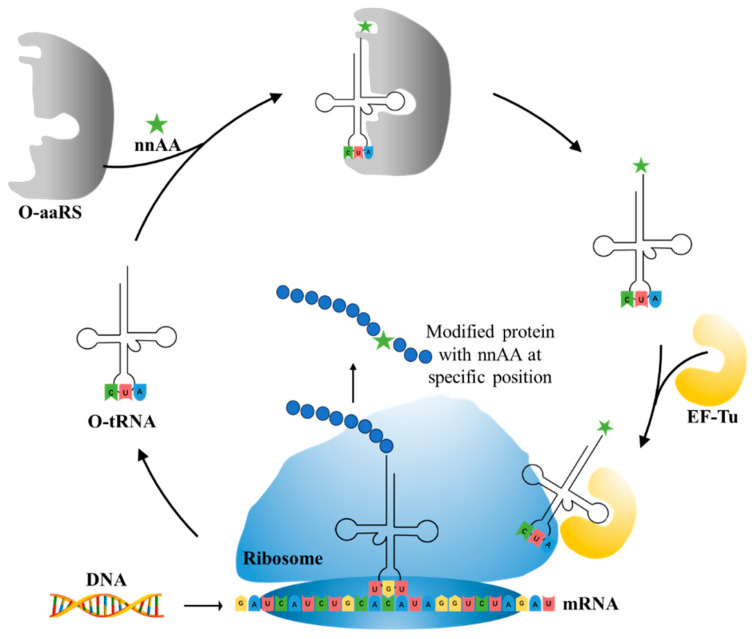
Scheme of site-specific incorporation of non-natural amino acids (nnAAs, represented by stars). O-aaRS represents orthogonal aminoacyl tRNA synthetase; nnAA represents non-natural amino acid; O-tRNA represents orthogonal tRNA; EF-Tu represents elongation factor Tu.

As shown in Figure 9, aminoacyl-tRNA synthetase (aaRS) uses specific anticodons to load amino acids onto transport RNA (tRNA), and then the tRNA charged with amino acids is delivered to the ribosome by elongation factor Tu (EF-Tu) for translation. The translation apparatus is crucial for the incorporation of nnAAs. The selective or specific incorporation of nnAAs into proteins first requires orthogonal aaRS-tRNA pairs. Ideal orthogonal aaRS/tRNA pairs do not cross-react with endogenous amino acids and aaRS/tRNA pairs of host cells but are recognized by host ribosomes [183], such as the orthogonal tyrosyl-tRNA synthetase TyrRS-tRNA_CUA_ pair from *Methanocaldococcus jannaschii*, the TyrRS-tRNA_CUA_ and LeuRS-tRNA_CUA_ pairs from *E. coli*, and the pyrrolysyl-tRNA synthetase PylRS-tRNA_CUA_ pairs from *Methanosarcina barkeri* and *Methanosarcina mazei* [184]. During the optimization process of orthogonal aaRS-tRNA pairs, researchers found that the editing domain of aaRS, responsible for hydrolyzing mismatched amino acids, is one of the key targets related to the incorporation efficiency of nnAAs. The T252Y mutation at the active site of the *E. coli* LeuRS enzyme editing domain reduces the editing activity of natural leucine, thus allowing for the introduction of leucine analogs oxonorvaline [185] and effectively increasing the incorporation efficiency of nnAAs. Later, Gan et al. [186] simultaneously evolved the anti-codon binding domain of aaRS and the amino acid binding pocket of EF-Tu and adjusted the expression of the evolved translation components in a single vector, further improving the incorporation efficiency of nnAAs. Given the low efficiency of traditional evolutionary techniques and the poor activity and selectivity of the evolved aaRS, Liu et al. [187] evolved a PylRS variant with a 45 times higher catalytic efficiency than that of the wild type and a TyrRS variant with increased selectivity for *p*-iodo-l-phenylalanine through phage-assisted continuous evolution (PACE).

In addition to orthogonal aaRS/tRNA pairs and elongation factors, ribosome and release factors (RFs) also significantly affect the incorporation efficiency and specificity of nnAAs. *O*-ribosomes containing O-16S rRNA and anti-Shine-Dalgarno (ASD) sequence mutations (for instance, 5′ GGAGG or 5′ CACAC) were introduced into *E. coli*. The *O*-ribosome could selectively translate orthogonal mRNAs, i.e., O-mRNAs, containing *O*-ribosome binding sites (such as SD sequence 5′ CCTCC or 5′ GTGTG) but could not translate native mRNA transcripts (SD sequence 5′ GGAGG) [188,189]. These modifications were mainly applied to the 16S rRNA of the orthogonal ribosome’s small subunit, while the 23S rRNA of the large subunit was shared by endogenous ribosomes and orthogonal ribosomes. The free exchange of subunits limited the development of orthogonal genetic systems. To further reduce the association with endogenous 16S or 23S subunits, Orelle et al. [190] produced a functional ribosome, Ribo-T, by engineering short rRNA linkers, by which the large and small subunits were covalently tethered into an entity. Because the linking subunits were associated without specificity and mediated translation by association with endogenous subunits, the activity of Ribo-T was still low. Utilizing tether libraries with different sequence lengths and compositions, a new oRibo-T v2/mRNA pair was optimized. Compared with Ribo-T, the growth rate of the new oRibo-T v2 system was increased by 86%, and multiple nnAAs could be incorporated into the synthesized peptides in a site-specific way [191]. Chin et al. also reported an *O*-stapled ribosome linked via the optimized RNA staple, which had a similar ability to support cell growth as a natural ribosome [192]. Nonetheless, the translation system needs to be further optimized to incorporate multiple different nnAAs into the protein simultaneously. With the maturation of genetic code expansion techniques, more blank codons could be used for nnAAs incorporation by reassigning sense or nonsense codons and introducing quadruplet codons and non-natural nucleotides [193], but natural ribosomes recognized tRNAs with quadruplet codons poorly [194]. Based on the orthogonal ribosome ribo-X [195], Chin and colleagues synthetically evolved ribo-Q1 that was able to take advantage of the quadruplet codons with a similar efficiency and fidelity as a triplet codon. In the presence of Seryl-tRNA synthetase variants/tRNA pairs and MbPylRS-tRNA_CUA_, ribo-Q1 successfully incorporated two different nnAAs into calmodulin GST-CaM-His_6_ [196].

Moreover, the incorporation of nnAAs via the reassignment of nonsense codons (mostly amber codons) was usually limited to peptide chain termination mediated by the release factor RF1. Mukai et al. [197] mutated the stop codon (UAG-UAA) of seven essential genes and then successfully deleted RF1; the incorporation efficiency of nnAAs with the amber suppressor tRNA was improved. It is worth mentioning that the addition of RF1-inhibiting antimicrobial peptide apidaecines into the expression medium could also promote the nnAAs entry into *E. coli* BL21 and DH10B without removing the RF1 gene [198].

As mentioned above, since each method of incorporating nnAAs (RSI and SSI) has its advantages and disadvantages, it is worth considering a combination of these two approaches—that is, employing orthogonal tRNA/synthetase pairs in autotrophic cells to carry out the site-specific incorporation of nnAAs while making use of endogenous tRNA synthetase to achieve the global incorporation of other nnAAs [199,200]. Furthermore, cell-free protein synthesis (CFPS) systems are also powerful tools for protein transcription and translation by the use of cell crude extracts or purified OTS components (PURE system) [201,202]. Since there is an open reaction environment lack of living cells, nnAAs can be directly added to the translation reaction without considering the toxicity of orthogonal tRNA/aaRS pairs, which is an important supplement to the above methods. In conclusion, to further improve the incorporation efficiency of nnAAs, the translation mechanism should be considered for coordination optimization as a complex system including codons, tRNAs, aaRSs, EF-Tu, and ribosomes.

### 5.3. Applications in Synthetic Biology

#### 5.3.1. Enzymes Engineering

Improving enzyme activity, stability, and stereoselectivity is a crucial aspect of enzyme engineering. Over the past two decades, numerous nnAAs have been successfully applied to enzyme modification to enhance activity and selectivity (Table 1). This has even enabled researchers to discover novel catalytic reactions that are not available in nature [203].

##### Enzyme Activity

ω-transaminase (TAms) is one of the most promising biocatalysts. It has been reported that the incorporation of several nnAAs could enhance the activity of TAm. The Phe88 residue at the active site of TAm was replaced by *p*-benzoylphenylalanine (*p*BzF), which reshaped the size of the active pocket while maintaining hydrophobicity. The resulting TAm variant exhibited significantly improved activity towards 1-phenylpropane-1-amine and benzaldehyde and also broadened the range of substrates [204]. After replacing tyrosine with 3-fluorotyrosine (mFY) by residue-specific incorporation, the synthesis activity of TAm for (*S*)-1-phenylethylamine was two time higher than that of the wild type in the presence of 20% DMSO (*v/v*) [205].

Because *p*BzF and other phenylalanine-derived nnAAs are easy to synthesize and have a high incorporation efficiency, they are widely applied in enzyme engineering. The Phe385 of transketolase (TK) played a key role in acceptor substrate binding, and after replacing it with a series of phenylalanine derivatives, the specific activity of the *p*-cyanophenylalanine (*p*CNF) variant to 3-hydroxybenzaldehyde (3-HBA) increased by 43 times [206]. Another example is *E. coli* nitroreductase (NTR), whose Phe124 was substituted by eight nnAAs such as *p*AMF, *p*BzF, *p*MF, *p*tfmF, and *p*NF, among which *p*NF-NTR had the highest activity against the substrate CB1954 or LH7. It was more than 30 times higher than the natural NTR and more than 2.3 times higher than the optimal natural NTR [207]. In a reported TEM-1 β-lactamase mutant library with a single nnAA substitution, the *p*-acrylamido-phenylalanine (AcrF) variant of valine-216 increased its catalytic efficiency by eight times [208]. Besides the residues in or near the active site, any changes in the protein structure, even away from the active site, might also have an impact on the catalysis. Substituting four phenylalanine residues that were not near a catalytic site or DNA binding site in restriction endonuclease *Pvu*II with nnAAs, the enzyme activity of *m*-fluorophenylalanine (mFF) variant was twice as high as that of the wild type [209]. Additionally, the incorporation of nnAAs was able to be used for the site-specific immobilization of enzymes, which could significantly improve the lifetime and maintain the activity of enzymes during biocatalytic applications. Smith et al. [210] specifically incorporated *p*-propargyloxy-phenylalanine (pPa) in a specific site of green fluorescent protein (GFP), and the modified GFP was covalent to the superparamagnetic bead via pPa. The immobilized GFP remained active and stable under harsh conditions, including repeated freeze–thaw and incubation at high temperatures in urea.

Along with widely used phenylalanine derivatives, analogs of methionine, cysteine, tyrosine, and histidine have also been shown to enhance enzyme activity. Substituting norleucine (Nle) for methionine in cytochrome P450 peroxidase [211] from *Bacillus megaterium* and in lipase [212] from *Thermoanaerobacter thermohydrosulfiricus*, the activity of the variant was increased by 2–10 times. The random incorporation of various sulfhydryl nnAAs into the active sites of *N*-acetylneuraminic acid lyase (NAL) resulted in significant increases in the activity of Phe190Dpc variants regarding the aldol condensation of erythrose and pyruvate [213]. Incorporating nnAA l-(7-hydroxycoumarin-4-yl)ethylglycine (Hco) into phosphotriesterase (arPTE) cloned from *Agrobacterium radiobacter* yielded a variant with an 8–11 times higher hydrolysis conversion rate, which was not obtained by natural amino acid optimization [214]. When *O*-methyltyrosine (*OMe*Tyr) was integrated into the residues Phe365 and Phe605 of the squalene-hopene cyclase active site, the enzyme activity was improved at low temperatures (<40 °C) [215]. Tyrosine analogs can also simulate natural post-translational modifications. Lu and colleagues incorporated 2-amino-3-(4-hydroxy-3-(1*H*-imidazol-1-yl) propanoic acid (ImiTyr) into sperm whale myoglobin, and the Tyr-His crosslinking required for the function of heme-copper oxidase (HCO) was mimicked [216]. The HCO functional model Mb_Phe33ImiTyr reduced oxygen three times faster than Mb_Phe33Tyr and released fewer ROS byproducts. In addition, Wang and colleagues introduced synthetic 2-amino-3-(4-hydroxy-3-(methylthio)phenyl)-propanoic acid (3-methylthiotyrosine or MtTyr) [217] to position 33 of myoglobin; the activity of hydroxylamine reductase in the mutant protein was increased.

Excitingly, the incorporation of nnAAs could lead to new protein functions. (2,2′-bipyridin-5-yl) alanine (BpyAla) with a copper ion chelating capacity was incorporated into a noncatalytic catabolite activator protein; in the presence of Cu (II) and 3-mercaptopropionic acid, the recombinant protein acquired the ability to catalyze double-stranded DNA breaking [218]. Using N_δ_-methyl histidine (NMH) as a non-classical catalytic nucleophile, a hydrolase capable of ester hydrolysis was prepared with the BH32 scaffold protein [219].

##### Enzyme Stability

Enzyme stability is a crucial factor for determining the practical application of biocatalysis. There are numerous reports on improving stability by the incorporation of nnAAs. Phosphotriesterase (PTE) exhibited a complete loss in structure at 70 °C for 15 min, whereas pFF-PTE incorporated with 4-fluorophenylalanine (pFF) retained its structure by about 30% and showed enhanced refoldability. The melting temperature (Tm) of pFF-PTE was increased by 1.3–2.5 °C [220]. Thus, fluorination nnAAs provided additional stability and protection against thermal inactivation. Likewise, replacing the tyrosine of ω-aminotransferase with 3-fluorotyrosine (mFY) significantly improved the thermal stability and organic solvent tolerance of the variant as well as the increased catalytic activity [205]. The variant retained 36% and 90% of the residual activity at 70 °C and in the presence of 50% (*v*/*v*) dimethyl sulfolone (DMSO), respectively, while the wild-type enzymes showed a corresponding residual activity of only 3.3% and 51%.

Different nnAAs may enhance protein stability in different ways. Thiol-containing nnAAs can form extended disulfide bonds (over ~11 Å) with Cys, breaking the restriction of conventional short disulfide bonds in natural amino acids (the bond length between two β-carbons is approximately 5.5 Å). The incorporation of thiol-containing nnAAs including *O*-(2-mercaptoethyl)-l-tyrosine (SetY), *O*-(3-mercaptopropyl)-l-tyrosine (SprY), and *O*-(4-mercaptoethyl)-l-tyrosine (SbuY) allows the melting temperature (Tm) of β-lactamase variants to increase without a loss of catalytic activity [221]. In the dimeric *E. coli* homoserine *O*-succinyltransferase (*metA*), the replacement of Phe21 by *p*BzF increased the melting temperature (74 °C) by 21 °C, improving the enzyme stability owing to strengthening the interaction between the monomers [222]. In another study, 13 kinds of nnAAs were introduced into the lipase (TTL) of *Thermoanaerobacter thermohydrosulfuricus*, and the results showed that the introduction of nnAAs conferred TTL protective effects against protein denaturant, alkylation, and inhibitory reagents [223]. Moreover, the addition of large halogenated nnAAs, i.e., 3-chloro-l-tyrosine and 3-bromo-l-tyrosine, to selected sites of glutathione *S*-transferase improved thermal stability [224] through the halogen molecules fulfilling the internal space and forming nonstandard stable interactions with neighboring residues.

##### Stereoselectivity and Regioselectivity

Controlling the stereoselectivity of biocatalysts is a challenging task in protein engineering, and nnAAs may provide a new dimension for achieving this task because some successful studies using nnAAs have been reported. Tryptophan is the largest natural amino acid, and the tryptophan residue at 222 of diketoreductase determines the substrate orientation. The incorporation of nnAAs with side chains larger than the tryptophan, such as *O*-tert-butyl-l-tyrosine (BuOF) and 4-phenyl-l-phenylalanine (BiF) at W222, enhanced enantioselectivity [225]. Specifically, wild-type enzymes showed an (*R*)-preference, with an enantiomeric excess (e.e.) of 9.1% for the substrate 2-chloro-1-phenylethanone, while the BuOF variant gave a higher e.e. of 33.7%. Similarly, the incorporation of BpyAla in the selected site of the transcription factor Lactoccocal multidrug resistance regulator (LmrR) could promote enantioselective Friedel–Crafts alkylation [226], and the e.e. of its products could reach 83%. Another example was the site-specific incorporation of the tyrosine analogs 3-(2-naphthyl)-alanine (NapA) into an engineered P450 enzyme variant called CYP102A1-139-3 at Ala328, which converted (*S*)-ibuprofen methyl ester into tertiary alcohol with 95% regioselectivity. By comparison, the parent enzyme produced a mixture of benzylic alcohol (62%) and tertiary alcohol (38%). The complete stereoselective oxidation of (+)-nootkatone to 9(*R*)-hydroxy-nootkatone was observed when Ala78 was replaced with *p*-acetyl-phenylalanine (pAcF), which was a novel reaction for wild-type enzymes [227].

**Table 1:** Non-natural amino acids mentioned in the review.

Abbreviations	nnAA	Structural Formula	Target Protein	Reference
*p*BzF	*p*-benzoylphenylalanine	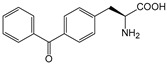	TAm	[204]
			*metA*	[222]
*p*CNF	*p*-cyanophenylalanine	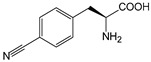	TK	[206]
*p*NF	*p*-nitrophenylalanine	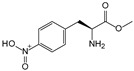	NTR	[207]
AcrF	*p*-acrylamido-phenylalanine	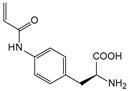	TEM-1 β-lactamase	[208]
pFF	4-fluorophenylalanine	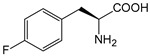	PTE	[220]
pPa	*p*-propargyloxy-phenylalanine	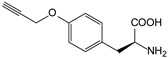	GFP	[210]
mFF	*m*-fluorophenylalanine	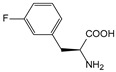	*Pvu*II	[209]
BiF	4-phenyl-l-phenylalanine	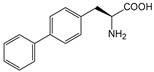	diketoreductase	[225]
BuOF	*O*-tert-butyl-l-tyrosine	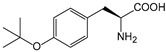		
SetY	*O*-(2-mercaptoethyl)-l-tyrosine	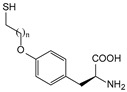 n = 1, SetY n = 2, SprY n = 3, SbuY	β-lactamase	[221]
SprY	*O*-(3-mercaptoethyl)-l-tyrosine			
SbuY	*O*-(4-mercaptoethyl)-l-tyrosine			
mFY	3-fluorotyrosine	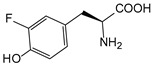	TAm	[205]
—	3-chloro-l-tyrosine	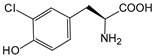	glutathione S-transferase	[224]
—	3-bromo-l-tyrosine	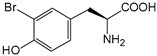		
ImiTyr	2-amino-3-(4-hydroxy-3-(1*H*-imidazol-1-yl) phenyl) propanoic acid	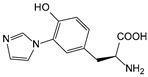	HCO	[216]
MtTyr	2-amino-3-(4-hydroxy-3-(methylthio) phenyl)-propanoic acid	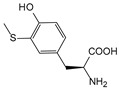	myoglobin	[217]
*OMe*Tyr	*O*-methyltyrosine	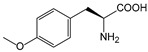	squalene-hopene cyclase	[215]
pAcF	*p*-acetyl-phenylalanine	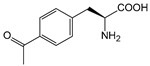	cytochrome P450 CYP102A1-139-3	[227]
NapA	3-(2-naphthyl)-alanine	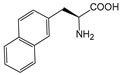		
BpyAla	(2,2′-bipyridin-5-yl) alanine	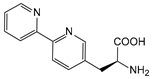	noncatalytic catabolite activator protein	[218]
			LmrR	[226]
Hco	l-(7-hydroxycoumarin-4-yl) ethylglycine	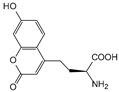	arPTE	[214]
Nle	norleucine	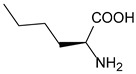	cytochrome P450	[211]
			lipase	[212]
Dpc	2,3-dihydroxypropyl cysteine	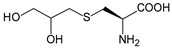	NAL	[213]
NMH	*N*_δ_-methyl histidine	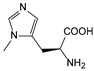	BH32 scaffold protein	[219]

#### 5.3.2. Cellular Process Controlling

Apart from enzyme engineering, some cellular processes can be controlled or modified with nnAAs. Phosphothreonine lyase, OspF, secreted by enteric pathogens, can remove the phosphate group of mitogen-activated protein kinase (MAPK), thus interfering with the MAPK pathway. By replacing the key catalytic residue Lys134 of OspF with nnAA, *N*^ɛ^-*p*-azidobenzyloxycarbonyl lysine (PABK), the variant lost dephosphorylation activity towards MAPK. However, with the addition of strained alkenes (*S*, *E*)-cyclooct-4-en-1-ol that can undergo a 1,3-dipolar cycloaddition reaction with PABK, the rescued OspF regained dephosphorylation activity and inhibited the MAPK signaling pathway, providing a valuable tool for manipulating the MAPK signaling pathway [228]. The method was also used to control the activity of Src kinase in HEK293T cells. Since most protein kinases contain a conserved lysine residue, the authors proposed the vision of applying this approach to dissect intracellular signaling networks. Similarly, nnAAs capable of undergoing palladium-catalyzed propargyl removal reaction can activate specific signaling pathways by activating proteins in living cells [229]. In addition, both chemical and photoinduced artificial genetic switches have been successfully established [230,231]. The switching mechanism involves introducing nnAAs with chemically unstable or photosensitive functional groups at critical sites of the protein, resulting in reduced protein activity or a loss of protein activity. Subsequently, specific small molecules or specific wavelengths of light are used to reactivate the protein. This method can also be used to regulate cascading signals in cells [232].

#### 5.3.3. Chassis Strain Engineering and Tracking

Microbial cell factories are important for the efficient production of various chemicals sustainably, but the imbalance between cell growth and product synthesis often leads to a reduced yield. nnAAs can be used to design chassis strains that produce functional products by balancing cell metabolism. In *E. coli*, using different concentrations of pAcF to regulate the balance of glycolysis and *N*-acetylglucosamine production resulted in a 4.54-fold titer improvement [233]. Similarly, regulating the expression of key genes in *Bacillus subtilis* with *OMe*Tyr led to a 2.34-fold increase in the titer of *N*-acetylneuraminic acid [233]. Moreover, the nnAA-dependent regulation of essential gene expression enabled engineered *Bacillus subtilis* to demonstrate effective biocontainment, which has been proposed to reduce the risk of genetically modified organisms in the natural environment. Another convincing example was the engineered strain in which the expression of the essential genes MurG, DnaA, and SerS depended on phenylalanine-derived nnAAs. After being cultured for 7 days on solid plates or for 20 days in liquid media, the strain grew well, and no escape frequency was detected [234]. This strategy has also been used to construct *E. coli* chassis strains depending on other nnAAs, such as NMH [235].

On the other hand, nnAAs also have good applications in tracking engineered bacteria in vivo. By introducing *p*-azido-l-phenylalanine (pAzF) into the cell surface protein (CsgA) variant of engineered probiotic *E. coli*, the engineered strain could be covalently labeled with Cy5 dye when pAzF was incorporated, and the CsgA displayed extracellularly, which enabled microorganisms tracking in the mice gastrointestinal tract [162]. Moreover, the fluorescence labeling of hidden epitopes of target transmembrane proteins in live neurons has been reported, utilizing the non-natural amino acid trans-cyclooctene derivatized lysine (TCO*A). And most excitingly, this labeling strategy could also be applied to cultured tissue slices, facilitating more in situ imaging of cells and tissues [236].

As mentioned above, the widespread and prominent applications of nnAAs have been developed in synthetic biology, but the high cost of non-natural building blocks remains a major obstacle to their industrial applications. The further development of pathways for synthesizing nnAAs in a simple and cost-effective manner will be a milestone in enzyme engineering and will also provide better opportunities for synthetic biology.

## 6. Summary, Challenge, and Perspective

The design and synthesis of artificial small molecules greatly enrich the toolbox of enzyme engineering. The incorporation of biological metal cluster mimics, mNADs, designer cofactors, XNAs, and nnAAs can not only improve enzyme activity, stability, and stereoselectivity but also lead to many novel catalytic reactions. These different artificial small molecules are mostly used for the modification of a certain type of enzyme. For example, biological metal cluster mimics are mainly used in metal enzyme engineering, while mNADs and designer cofactors are mostly applied in oxidoreductases. In comparison, XNAs and nnAAs have a broader coverage for enzyme engineering. Despite the widespread and successful applications of artificial small molecules, some challenges such as a high production cost and low incorporating efficiency have yet to be conquered.

The traditional synthesis methods for artificial small molecules often involve the issues of complex reactions, high costs, and environmental pollution. Therefore, developing green and efficient biological synthesis methods is of great significance. A representative successful example is the biosynthesis of non-natural coenzyme NCD [6] and NMN [7,8]. However, most artificial small molecules have not yet been completely biosynthesized. For nnAAs, there have been more advances in semi-synthesis that require an exogenous supplement of intermediate substrates. For example, by modifying the in vivo synthesis pathway of cysteine and adding aromatic thiols exogenously, nearly 50 kinds of nnAAs have been biosynthesized [237]. In contrast, rare nnAAs—mainly p-aminophenylalanine [238], *O*-phospho-l-threonine [239], and 5-hydroxytryptophan [240]—have been reported to be completely de novo synthesized from simple carbon sources. It can be seen that the in vivo biosynthesis of nnAAs remains challenging: the currently defined metabolic pathways, some of which lack information about key enzymes, only cover the synthesis of a small number of nnAAs, while the biosynthesis pathways for most nnAAs are unknown or do not exist; meanwhile, some nnAAs will inhibit the growth of chassis strains during biosynthesis and may encounter difficulties in scaling up during fermentation. Thus, the titer of biosynthesized nnAAs may be insufficient. Future developments are needed to overcome these problems by integrating the technologies of synthetic biology, computational biology, chemical biology, and protein science.

Another bottleneck is the incorporation efficiency of artificial small molecules. Natural enzymes have generally evolved to incorporate the native cofactors with the best efficiency. Therefore, to achieve a high incorporation efficiency, the enzymes should be engineered together with the artificial small molecule. Co-engineering of the small molecule and the protein has been successfully achieved for the artificial P450 enzyme containing DFSM [146]. These successful studies provide promising directions for future artificial enzyme development.

In addition, we also expect that different categories of artificial small molecules can be used in combination with each other. Based on previous reports on hydrogenase modification [241], where metal clusters were anchored by non-natural amino acids, we speculate the possibility of combining site-specific incorporation methods of nnAAs with biological metal cluster mimics to modify target proteins. This can anchor metal cluster mimics or other designer cofactors at the expected sites. The synthesis of NAD analogs based on proteogenic amino acids [131] has also opened up new possibilities for the application of nnAAs in the design of mNADs and designer cofactors.

Another technology that could potentially drive the development of artificial small molecules is artificial intelligence (AI). AI has already played an important role in enzyme engineering and chemical catalysts [242,243,244], while its application in small-molecule cofactors is rarely reported. One reason is that there are not enough successful cases of cofactor engineering to accumulate sufficient data. Another reason is that cofactor engineering needs to be combined with protein engineering, which further increases the difficulty. In the future, with the increase in experimental data and the advancement of AI technology, great progress is expected in the better design and synthesis of artificial cofactors. Laboratory automation provides a more efficient solution for testing these cofactors, and the successful development of artificial cofactors will inevitably increase our knowledge of the synergistic catalysis between enzymes and small molecules. Driven by the DBTL (Design–Build–Test–Learn) cycle of synthetic biology, it is expected that enzymes, cells, and even organisms that meet industrial requirements will be designed and constructed in the future. The design and development of artificial small-molecule cofactors and building blocks are quite essential in this process.

## Data Availability

No new data were created or analyzed in this study. Data sharing is not applicable to this article.
